# Concentrate Supplement Modifies the Feeding Behavior of Simmental Cows Grazing in Two High Mountain Pastures

**DOI:** 10.3390/ani8050076

**Published:** 2018-05-16

**Authors:** Alberto Romanzin, Mirco Corazzin, Edi Piasentier, Stefano Bovolenta

**Affiliations:** Department of Agricultural, Food, Environmental and Animal Sciences, University of Udine, 33100 Udine, Italy; alberto.romanzin@uniud.it (A.R.); edi.piasentier@uniud.it (E.P.); stefano.bovolenta@uniud.it (S.B.)

**Keywords:** grazing behavior, alpine pasture, supplement, automatic feeding behavior recorder

## Abstract

**Simple Summary:**

Traditional Alpine husbandry systems require dairy cows to be grazing on mountain pasture during summer and kept indoors during the remaining part of the year. Nowadays, the pasture is not able to fully satisfy the nutritional requirements of cattle; therefore, the use of concentrates is frequently required. From their use, some issues arise: the cows tend to consume the concentrates at the expense of the grass; concentrates are competitive with human diets; concentrates decrease the environmental sustainability of farm. Therefore, in order to minimize their use, it is imperative to obtain data on the grazing behavior of cows. The aim of this study was to assess the effect of concentrate levels on the behavior of dairy cows during summer grazing in two pastures characterized by *Poion alpinae* and *Seslerion caeruleae* alliance. Cows were equipped with an electronic device to evaluate feeding behavior (grazing, rumination, and walking). In addition, the plant selection by animals was assessed. In *Poion alpinae*, a rich pasture, the increased supplement influenced the selectivity of the pasture species, while in *Seslerion caeruleae*, a poor pasture, supplementation resulted in a reduction in grazing times. The study highlights how the supplement level induced a different grazing behavior depending on pasture type.

**Abstract:**

During grazing on Alpine pastures, the use of concentrates in dairy cows’ diet leads to a reduction of the environmental sustainability of farms, and influences the selective pressure on some plant species. In order to minimize the use of concentrates, it is imperative to obtain data on the grazing behavior of cows. The aim of this study was to assess the effect of concentrate levels on the behavior of dairy cows during grazing. One hundred and ten lactating Italian Simmental cows, that sequentially grazed two pastures characterized by *Poion alpinae* (Poion) and *Seslerion caeruleae* (Seslerion) alliance, were considered. For each pasture, eight cows were selected and assigned to two groups: High and Low, supplemented with 4 kg/head/d, and 1 kg/head/d of concentrate respectively. Cows were equipped with a noseband pressure sensor and a pedometer (RumiWatch system, ITIN-HOCH GmbH) to assess grazing, ruminating, and walking behavior. In addition, the plant selection of the animals was assessed. On Poion, increased supplement intake caused a more intense selection of legumes, without affecting feeding and walking times. On Seslerion, grazing time was higher in Low than High. Grazing management in alpine region must take into account the great variability of pastures that largely differ from a floristic and nutritional point of view.

## 1. Introduction

The traditional farming system of dairy cows in the Alps requires animals kept in valley floor stables from autumn to spring and in mountain pasture during summer [1]. In this period the animals, usually belonging to local dual-purpose breeds, are in late lactation because calvings are concentrated in the autumn and winter [2]. Alpine pastures are used sequentially according to vegetation gradient, enabling effective utilization of forage mountain resources [3], and potentially improving animal welfare [4,5].

In recent decades, the gradual intensification of the agricultural sector, also in the mountains, has increased the production performance of local breeds. In many cases, these breeds have been replaced with others of high genetic merit, such as the Holstein Friesian, and the calvings are now distributed throughout the year [2].

Genetic improvement of dairy cow breeds has led to a broad increase in the nutritional requirements of animals that can no longer be satisfied by grazing alone [6,7], because of the moderate herbage yields of alpine pastures. Therefore, it is increasingly necessary to properly supplement the cows’ diet, even on the pastures, to produce milk yields close to the genetic potential of the breed, and/or to ensure appropriate animal body conditions are maintained. However, this necessity could involve nutritional and agroecological problems. From a nutritional point of view, the objective of covering these requirements is very difficult to achieve, because according to the substitution rate mechanism [8,9], supplementation may reduce herbage intake and feeding time on pasture, and influence the selective pressure on certain pasture plant species [10]. From an agroecological point of view, concentrates often represent an external input for the mountain farm, reducing its environmental sustainability [11], and are also in competition with human nutrition [12]. In order to minimize their use, it would be very useful for the breeder to obtain data on the grazing behavior of dairy cows as a direct or indirect result of different feeding management strategies.

In the past, several studies were carried out on the impact of feed supplementation [13,14,15,16], but also of stocking rate [17,18] and herd management [19] on grazing behavior. In general, these studies evaluated herbage intake, grazing time, and the walking times of animals, but the methods utilized (e.g., the double marker methods for the herbage intake estimation or the visual techniques for the grazing behavior assessment) were burdensome, time consuming, and feasible only in experimental contexts. However, many efforts have been made to provide easier and more reliable methods and tools to evaluate the behavior of grazing ruminants. In particular, compact electronic equipment for continuous monitoring of feeding and locomotion behavior provide reliable data at ever lower costs [20]. RumiWatch system is a sensor monitoring system widely used indoors [21,22]. Despite the fact that it was recently proposed for grazing assessment [23,24], to our knowledge, no papers have reported its application in evaluating the effect of different management strategies on feeding behavior.

The aim of the present study is to evaluate grazing, rumination, and the walking behavior of dairy cows receiving different levels of concentrates, through the use of this monitoring system. In addition, the plant selection by grazing animals was assessed.

## 2. Materials and Methods

The trial was carried out in accordance with EU Directive 2010/63/EU; it complied to the Italian legislation on animal care (DL n. 26, 4 March 2014), and adhered to the internal rules of University of Udine.

### 2.1. Experimental Design and Treatments

The experiment was carried out in an alpine farm (Malga Montasio, Udine, Italy; 46°24′45″ N, 13°25′53″ E; 1500–1800 m a.s.l.), in two grazing periods of 20 days each. In the first grazing period (starting at 16th June), a herd of 110 lactating Italian Simmental cows was allowed to graze on a pasture located on average at 1520 m a.s.l., and referable to the *Poion alpinae* alliance (Poion). In the second one (starting at 21st July), the same herd grazed a pasture located at an average altitude of 1680 m a.s.l., and referable to the *Seslerion caeruleae* alliance (Seslerion). The experimental pastures were grazed at the same phenological stage (flowering period of gramineae). Cows were maintained day and night on pasture.

For each grazing period, eight multiparous dairy cows were selected and assigned to two balanced groups, according to milk yield (17.4 ± 0.7 kg), and days in milk (147 ± 9 d). For 10 days (pasture adaptation) the animals received a concentrate level (2.0 kg/head/d on average) according to milk yield. All the cows involved in the trial had already grazed for at least one season in the alpine farm used for the experiment. For the next 10 days (experimental period), four cows were supplemented with 4 kg/head/d of concentrate (High), while the other four cows were supplemented with 1 kg/head/d of the same concentrate (Low). The concentrate was based on corn, barley and soybean meal, beet pulp, and wheat bran. It was given twice a day during milking. During the experimental periods, the eight monitored cows continued to graze and to be milked with the herd.

### 2.2. Measurements

#### 2.2.1. Weather Conditions

Over the entire experimental period, weather conditions were recorded with an automatic weather station located between the two experimental pastures, at approximately 1600 m of altitude. The meteorological parameters recorded were precipitation, solar radiation, relative humidity, wind speed, and temperature.

#### 2.2.2. Pastures, Herbage and Concentrate Characteristics

For each pasture, at the beginning of the experimental period, the quantity and the composition of available herbage (AH) was estimated by cutting 6 strips of 10 m × 0.10 m at 4 cm stubble height, using electric grass shears (herbage mass available: Poion: 2.39 tons dry matter (DM)/ha, Seslerion: 1.43 tons DM/ha on average). The composition of selected herbage (SH) by dairy cows was estimated by the hand-plucking technique [25] during the last six days of experimental periods. Samples of AH and SH were hand-separated into Grasses, Legumes and other families. Grasses included some graminoid species belonging to the botanical families *Poaceae*, *Cyperaceae*, and *Juncaceae*. Herbage samples were oven-dried at 70 °C for 48 h. Botanical assessment, on a dry weight basis, was carried out on the sub-samples. The palatability index (PI) was calculated, for each sub-sample, as a ratio of the incidence in SH against their occurrence in AH [26]. Herbage and concentrate samples were analyzed for crude protein and ether extract, according to AOAC International [27], and for fiber fractions (NDF, ADF, ADL), according to Goering and Van Soest [28]. The nutritive value, expressed in feed units for milk (FUM), was estimated according to the French National Institute for Agricultural Research (INRA) standards [29]. The concentrate had 12.5% crude protein, 2.4% ether extract, 4.4% ash and 1.05 FUM/kg DM.

#### 2.2.3. Milk Production

Milk yields were recorded and individual samples were collected on the last two days of each experimental period, during evening and morning milking. The following determinations were made on milk samples: fat, protein and lactose [30], urea [27] and somatic cell count (SCC; Foss-o-Matic, FossElectric, Hillerod, Denmark). SCC data was analyzed as somatic cell score (SCS) = log_2_ (SCC/100000) + 3 [31].

#### 2.2.4. Cows Behavior

During the two experimental periods, the selected dairy cows were equipped with a noseband pressure sensor and a pedometer (RumiWatch system, ITIN-HOCH GmbH, Liestal, Switzerland), validated for use respectively by Ruuska et al. [32] and Alsaaod et al. [33], in order to assess feeding and locomotion behavior. At the end of each experimental period, raw data, automatically saved on an internal storage device during the last six days of the experimental period, was downloaded and analyzed with theRumiWatch Converter 0.7.4.10 (ITIN-HOCH GmbH, Liestal, Switzerland). The following variables of feeding behavior were calculated: grazing and rumination time (min/day), head-down time (min/day), number of grazing and rumination bites (n/day), number of rumination boli (n/day), grazing, rumination intensity (n bites/min and n bites/bolus), and activity index (without dimension, related to the variability of noseband tri-axial accelerometer data output). The following variables of locomotion behavior were calculated: walking and standing time (min/day), number of steps (n/day), and activity index (without dimension related to the variability of pedometer tri-axial accelerometer data output). To ensure that the data of feeding behavior were exclusively referring to grazing, the behaviors recorded during milking time of each cow were removed.

### 2.3. Statistical Analysis

All statistical analyses were performed using SPSS for Windows (version 7.5.21, SPSS Inc., Chicago, IL, USA). The normality of data distribution was tested using the Shapiro-Wilk tests, and, when appropriate, variables were transformed for parametric testing. The two pastures were separately analyzed. The effect of the supplement level on chemical and floristic composition of selected plant species, on milk yield and composition, and on daily feeding and locomotion behavior of animals was evaluated with a mixed model for repeated measures, as suggested by Wang and Goonewardene [34], considering supplement level as fixed factor and the day of grazing or of sampling as a repeated factor. In addition, the interaction supplement level × day was considered, but not reported because it never reached a level of significance. The effect of the supplement level on the hourly behavior of animals was evaluated with a mixed model for repeated measures, as suggested by Wang and Goonewardene [34], considering supplement level, day of grazing, and the hour of the day as fixed, block and repeated factor respectively, while animal was treated as random factor. In addition, the interaction supplement level × hour of the day was considered. If this interaction was significant, the differences between experimental groups were tested with unpaired *t*-test at specific hour of the day, as suggested by Park et al. [35]. A probability level of *p* ≤ 0.05 was considered significant, whereas 0.05 < *p* < 0.10 was considered a tendency.

## 3. Results and Discussion

### 3.1. Weather Conditions

**Figure 1:**
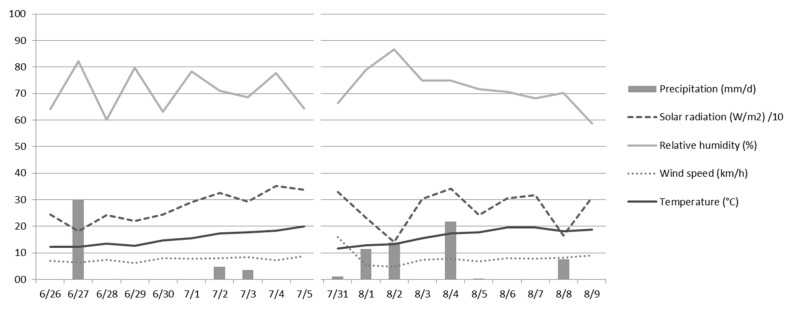
Weather conditions during the two experimental periods.

The weather conditions during the experimental periods are reported in Figure 1. The average environmental temperature fell within the range of thermoneutral zone of dairy cows [36]. Indeed, in the first and second grazing periods showed averages of 15.4 °C (min 6.9 °C, max 25.0 °C) and 16.4 °C (min 7.0 °C, max 25.7 °C) respectively. The wind remained between gentle and moderate, with only one day of strong breezes (daily wind velocity 21.8 km/h on average). The average precipitation was 3.9 mm/d and 5.6 mm/d, respectively, for the first and second experimental periods.

It is well known that weather conditions may affect the behavior of grazing cows, particularly wind, heavy rainfall, high temperatures, and intense solar radiation [37,38,39]. The weather conditions recorded during the two experimental periods did not seem likely to significantly alter the feeding behavior of cows.

### 3.2. Pasture Characteristics and Herbage Selection by Animals

The floristic composition of AH (Table 1) of Poion showed a high presence of Grasses (67.7% DM), mainly composed by *Poa alpina*, *Phleum rhaeticum*, *Festuca pratensis*, *Festuca rubra* ssp. *commutata* and *Helictotricon pubescens*. Legumes (4.0% DM) essentially comprised clovers (*Trifolium repens* and *Trifolium pratense*). Other families (28.4% DM) included several species widespread in *Poion alpinae* alliance as *Ranunculus acris*, *Alchemilla vulgaris*, *Plantago atrata*, *Veratrum album*, *Thymus praecox* spp. *polytrichus*, *Potentilla aurea* and *Veronica chamaedrys*. Grasses were 26.2% pp lower in SH than AH, without a significant difference between the experimental groups (*p* > 0.05). Conversely, Legumes were more present in SH than AH, and High showed greater values than Low (*p* < 0.05). It is well known that Legumes have a higher protein content and a greater palatability than Grasses [40]. However, the results of the herbage chemical analysis (Table 2) showed no significant differences between High and Low groups on Poion. The nutritional value of AH was 0.66 FUM/kg DM. Dairy cows selected the herbage in such a way as to increase the nutritional value of *ingesta* (0.79 FUM/kg DM on average).

Even AH of Seslerion showed a floristic composition (Table 1) characterized by a high presence of Grasses (60.6% DM), mainly composed by *Sesleria caerulea*, *Festuca rubra* ssp. *commutata*, *Carex sempervirens*, *Poa alpina* and *Koeleria pyramidata*, and a low percentage of Legumes (3.8% DM), mainly composed by *Anthyllis vulneraria* and *Lotus alpinus*. Other families (35.4% DM) included several species widespread in *Seslerion caeruleae* alliance, such as *Prunella grandiflora*, *Rhinantus glacialis*, *Potentilla crantzii*, *Polygonum viviparum*, *Helianthemum alpestre*, *Betonica alopecurus*, and *Acinos alpinus*. Unlike Poion, in Seslerion the cows consumed a large amount of Grass. In fact, this botanical family was reduced in SH to only 8.2 pp on average. This is also evident, considering PI values which are close to the unit, indicating that the Grass consumed approaches the Grass availability. The ability by animals to select different plant species was probably limited by the lower availability of herbage in this pasture. This evidence is supported by the chemical composition (Table 2) of SH, which is very similar between the experimental groups, and in turn, similar to AH. Even the nutritional value of the herbage was very similar between AH (0.65 FUM/kg DM) and SH (0.64 FUM/kg DM on average).

### 3.3. Milk Production and Composition

During grazing on Poion, the fat corrected milk (FCM), fat, protein, lactose, and SCS were 16.0 kg, 3.96%, 3.16%, 4.70% and 2.39 respectively, without differences between experimental groups (*p* > 0.05). During grazing on Seslerion, the FCM, fat, protein, lactose, urea, and SCS were 18.4 kg, 3.60%, 3.02%, 4.61%, 30.1 mg/dL and 3.97 respectively, without differences between experimental groups (*p* > 0.05). The milk composition is similar to previous studies [41]. Only milk urea concentration in Poion tended to be lower in High than Low (14.2 vs. 19.0 mg/dL; *p* = 0.09). Urea is an indicator of the nutritional status of animals, and its concentration in milk is in close correlation with the protein-energy ratio of the diet [42]. The mean values observed in this study were within the range proposed by Bendelja et al. [43], i.e. between 15–30 mg/dL, which correspond to normal levels of energy and protein in animals’ diets. The only exception was the low concentration observed in High on Poion pasture. In this case, the higher level of concentrate offered to animals has led to a slight alteration of the protein-energy ratio of the diet, with a consequent reduction of 25% in milk urea in comparison to High.

### 3.4. Feeding and Locomotion Behavior

Results concerning feeding and locomotion behavior are shown in Table 3. With regard to Poion, no statistical differences among experimental groups were observed (*p* > 0.05). These results are not in agreement with Bovolenta et al. [44] that, in an experiment carried out on Brown cows supplied with different amounts of supplement, had observed significant reductions in grazing time, and also in herbage intake. In the present study, 537 min/day of grazing time with a grazing intensity of 65 bites/min were recorded. This last value is similar to that found by Abrahamse et al. [45], and higher than values reported by O’Driscoll et al. [46] (64.5 and 58.5, respectively). These studies, carried out on Holstein Friesian cows grazing day and night, also reported comparable values for rumination times, which in our study were 473 min/day for both groups. It is well known that the level of fiber in the diet influences rumination behavior. Therefore, as expected, the rumination intensity (67 bites/min and 53 bites/bolus) and the number of boli (540) were similar. No significant difference was observed in terms of walking behavior (*p* > 0.05).

Concerning the grazing behavior on Seslerion, group Low grazed for more time (*p* < 0.01) than group High, probably due to low nutritional level of Seslerion, that would not be sufficient to meet the nutritional requirements of Low cows, which have probably compensated by grazing for longer times. This difference is reflected in total grazing bites per day: higher for Low of nearly 5000 bites (*p* < 0.05). Group Low has grazed with greater intensity (71 vs. 66 bites/min), although the difference did not reach statistical significance. No significant differences were observed in terms of rumination and walking behavior. The rumination intensity is slightly higher (69 bites/min and 56 bites/bolus) than the values reported in the two above-mentioned studies [45,46], probably because of the high level of fiber in Seslerion (Table 2).

In this trial, a direct measure of the animals’ DM intake was not available; however, it can be assessed following the regression equation developed by Vazquez and Smith [47] for grazing dairy cows. In the present study, the average DM intakes of herbage resulted 12.8 and 15.4 kg/d on Poion, and 12.7 and 15.1 kg/d on Seslerion for High and Low group respectively. Considering the concentrate intake, the total DM intakes were 16.4 and 16.3 kg/d on Poion, and 16.3 and 16.0 kg/on Seslerion for High and Low group respectively, indicating a substitution effect between herbage intake and concentrate. Taking into account the nutrition value of feedstuffs and the nutritional requirements of animals [29], the energy balance of dairy cows was different in the two experimental pastures. In Seslerion, the energy balance of cows was negative both in High and Low (−2.2 and −3.7 FUM respectively), and therefore, not able to maintain milk production. In Poion, the energy balance of cows was positive both in High and Low (+0.6 and +0.4 FUM respectively), because the animals had a predilection for plants with higher nutritional value. In this pasture, the Low group had a similar energy balance than the High, probably because the former had a more intensive selection of plants, as suggested by the tendentially higher (*p* = 0.087; Table 2) nutritional value of SH compared to the High group. This statement seems not to be in agreement with the values reported in Table 1 for the presence of Legumes in SH, probably because of the high presence of “other families”, around 50% of the herbage intake by animals, in SH.

**Figure 2:**
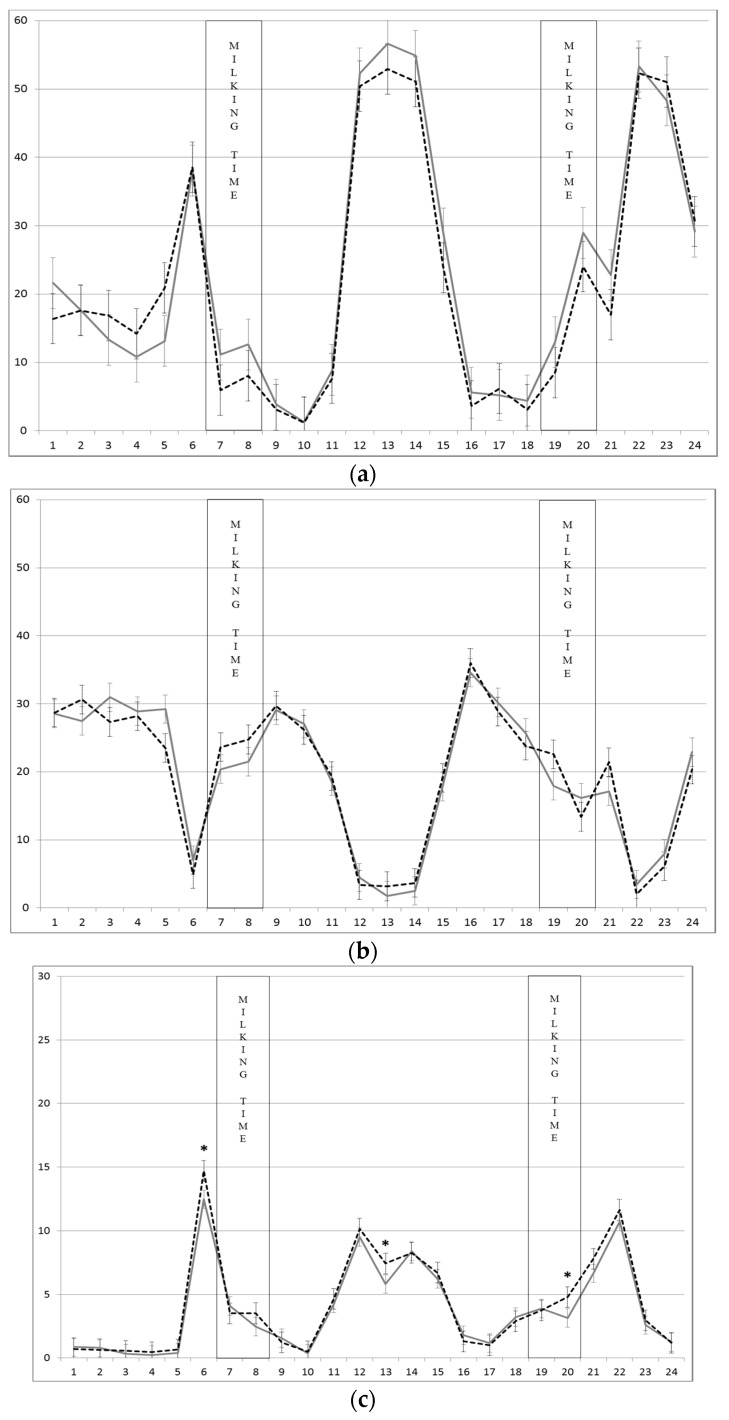
Mean ± SEM of (**a**) grazing time, (**b**) rumination time, (**c**) walking time (min/h) of dairy cows on Poion pasture supplemented with two levels of concentrates (High: solid line and Low: dotted line). The vertical bands represent the milking time of the whole herd. * *p* < 0.05.

Figure 2a–c show the hourly patterns of grazing, rumination, and walking time on Poion, respectively. The interaction supplement level × hour was significant (*p* < 0.01) for all these variables. In the first figure, there are three grazing sessions: the middle part of the day (main session), before the morning milking, and after the evening milking (secondary sessions). The rumination behavior prevails in the night and near milkings. These trends in grazing and rumination behaviors are in line with those found in other studies [44,48]. The distribution of the walking time (Figure 2c) was substantially related to two factors: moving from milking parlour to the pasture, and moving around the pasture during grazing.

**Figure 3:**
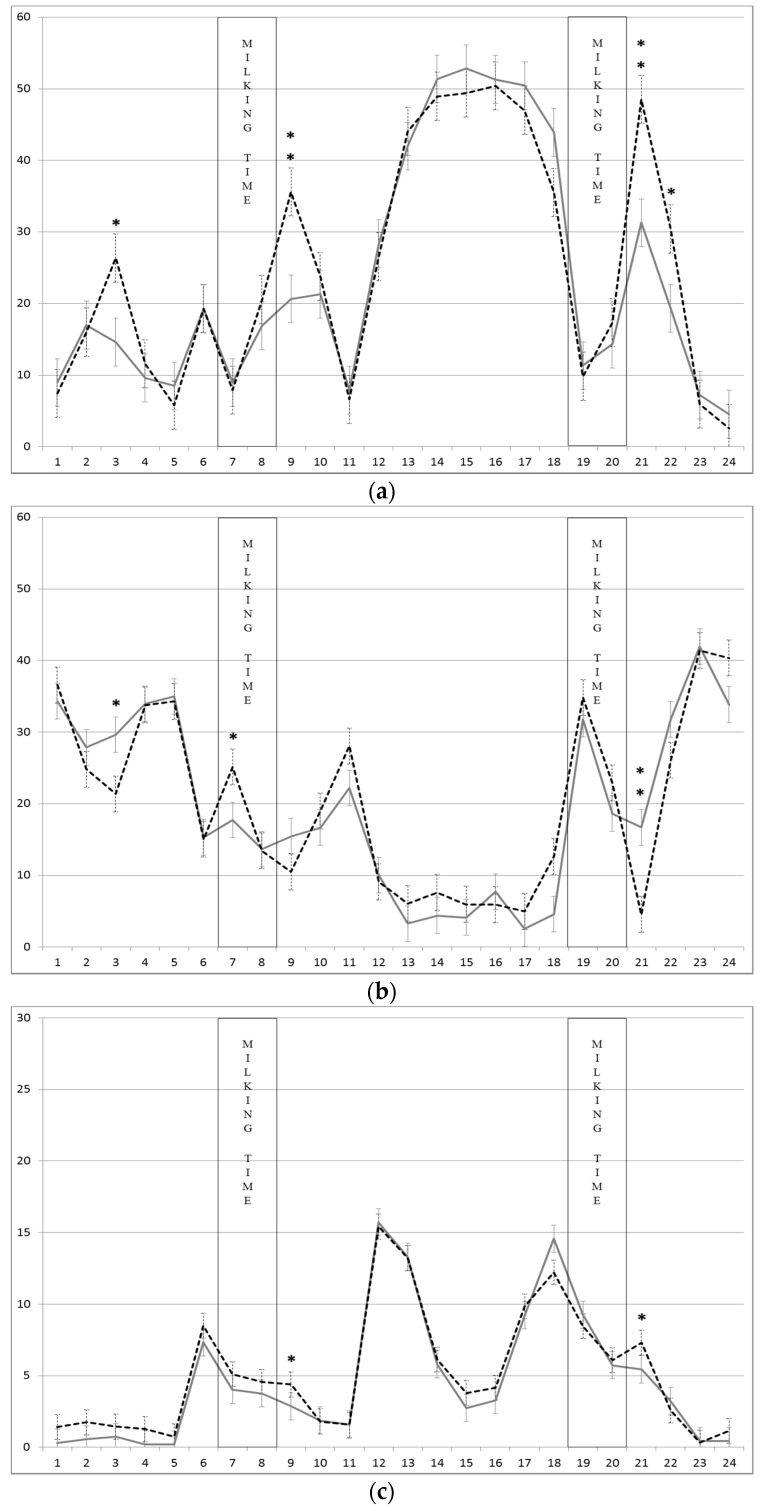
Mean ± SEM of (**a**) grazing time, (**b**) rumination time, (**c**) walking time (min/h) of dairy cows on Seslerion pasture supplemented with two levels of concentrates (High: solid line and Low: dotted line). The bands represent the milking time of the whole herd. ** *p* < 0.01; * *p* < 0.05.

Hourly patterns of grazing, rumination and walking time on Seslerion are shown in Figure 3a–c. The interaction supplement level × hour was significant (*p* < 0.01) for all these variables. Figure 3a allows an understanding of the times that have mainly contributed to the difference in the total grazing times shown in Table 3. On Seslerion, the main grazing session is longer than on Poion (from 11:00 h to 18:00 h). At this phase, excluding walking, the cows grazed all the time at their disposal. Nevertheless, in three sessions the Low group grazed significantly more than High group (03:00 h: *p* < 0.05; 09:00 h: *p* < 0.01; 21:00 h: *p* < 0.01; 22:00 h: *p* < 0.05). In two of these sessions, while the Low group grazed, the High group had ruminated for more time (03:00 h: *p* < 0.05; 21:00 h: *p* < 0.01), albeit without affecting the total daily rumination time. As far as walking time is concerned, peaks correspond to phases of more intensive grazing or movement from pasture to milking parlour and vice versa.

## 4. Conclusions

In alpine dairy farms, pastures are sequentially grazed according to the vegetative gradient. Breeders should take into account that different pastures can present significant differences from a floristic and nutritional point of view. This study has highlighted how the choice of two levels of supplementation induced a different grazing behavior on two experimental pastures. In Poion the highest level of concentrate offered to the grazing cows had a more marked effect on the selectivity, while in Seslerion this highest level resulted in a reduction in grazing times.

## Figures and Tables

**Table 1:** Floristic composition (% DM) of available herbage on pastures (AH; *n* = 6), selected herbage by the cows (SH; *n* = 48), and palatability index (PI), as affected by different concentrate levels (High and Low).

Pasture	AH	SH		SEM	*p-*Value *	PI		SEM	*p-*Value *
		High	Low			High	Low		
Poion									
Grasses	67.7	42.1	41.0	2.70	0.854	0.63	0.61	0.040	0.826
Legumes	4.0	9.8	6.3	0.54	0.032	2.40	1.63	0.150	0.035
Other families	28.4	48.1	52.6	2.77	0.445	1.81	1.94	0.109	0.640
Seslerion									
Grasses	60.6	55.8	49.0	2.93	0.153	0.92	0.81	0.032	0.128
Legumes	3.8	4.3	3.7	0.58	0.764	2.15	1.57	0.392	0.469
Other families	35.4	39.6	46.7	2.82	0.101	1.12	1.34	0.056	0.099

DM = dry matter; SEM = standard error of the mean; * the differences between High and Low were statistically tested.

**Table 2:** Chemical composition (% DM) and nutritive value (FUM/kg DM) of herbage consumed by cows in two pastures (Poion and Seslerion), as affected by different concentrate levels (High and Low).

Herbage	Poion			SEM	*p*-Value *	Seslerion			SEM	*p-*Value *
	AH	SH				AH	SH			
		High	Low				High	Low		
Chemical composition										
Crude protein	8.6	10.0	10.0	0.087	0.905	10.9	9.9	10.1	0.146	0.663
Ether extract	2.7	2.6	2.7	0.032	0.056	2.9	3.0	3.1	0.073	0.831
Ash	4.7	5.7	6.1	0.180	0.366	5.9	6.1	6.0	0.138	0.693
NDF	58.9	60.0	57.4	0.809	0.169	64.8	62.1	61.2	0.780	0.438
ADF	38.6	32.2	30.1	0.515	0.079	39.3	40.0	40.1	0.299	0.885
ADL	12.4	11.3	11.8	0.41	0.588	12.1	13.3	13.4	0.495	0.912
Nutritive value	0.66	0.77	0.81	0.010	0.087	0.65	0.64	0.63	0.005	0.919

DM = dry matter; FUM = feed units for milk; AH = available herbage; SH = selected herbage; SEM = standard error of the mean; * the differences between High and Low were statistically tested; NDF = neutral detergent fibre; ADF = acid detergent fibre; ADL = acid detergent lignin.

**Table 3:** Feeding and locomotion behavior of dairy cows grazing on two pastures (Poion and Seslerion) as affected by different concentrate levels (High and Low).

Items	Poion		SEM	*p*-Value	Seslerion		SEM	*p*-Value
	High	Low			High	Low		
Grazing time (min/d)	552	522	13.3	0.301	563	601	6.4	0.008
Grazing bites (n/d)	40,155	37,759	1271	0.380	41,084	45,959	1342	0.025
Grazing intensity (n bites/min)	65.1	64.9	1.35	0.961	66.4	70.8	1.05	0.065
Rumination time (min/d)	473	473	13.7	0.996	473	484	10.2	0.608
Rumination bites (n/d)	28,715	28,717	1002	0.999	28,975	31,315	974	0.275
Boli (n/d)	535	544	28.0	0.869	506	552	14.0	0.148
Rumination intensity (n bites/min)	66.8	67.2	1.46	0.902	67.0	71.7	1.51	0.171
Rumination intensity (n bites/bolus)	53.9	52.0	1.53	0.546	56.5	56.2	1.67	0.923
Walking time (min/d)	91	99	6.3	0.527	114	119	6.0	0.662
Standing time (min/d)	762	751	27.1	0.839	759	834	25.7	0.202
Steps (n/d)	3281	3535	202.4	0.551	3802	4355	199.4	0.212

SEM = standard error of the mean.
